# Statistical Evaluation of Total Expiratory Breath Samples Collected throughout a Year: Reproducibility and Applicability toward Olfactory Sensor-Based Breath Diagnostics

**DOI:** 10.3390/s21144742

**Published:** 2021-07-11

**Authors:** Katsushige Inada, Hiroshi Kojima, Yukiko Cho-Isoda, Ryo Tamura, Gaku Imamura, Kosuke Minami, Takahiro Nemoto, Genki Yoshikawa

**Affiliations:** 1 Department of Medical Oncology, Ibaraki Prefectural Central Hospital, Kasama 309-1793, Ibaraki, Japan; k-inada@chubyoin.pref.ibaraki.jp (K.I.); h-kojima@chubyoin.pref.ibaraki.jp (H.K.); y-chou@chubyoin.pref.ibaraki.jp (Y.C.-I.); 2 Ibaraki Clinical Education and Training Center, University of Tsukuba Hospital, Kasama 309-1793, Ibaraki, Japan; 3 World Premier International (WPI) Research Center for Materials Nanoarchitectonics (MANA), National Institute for Materials Science (NIMS), Tsukuba 305-0044, Ibaraki, Japan; TAMURA.Ryo@nims.go.jp (R.T.); IMAMURA.Gaku@nims.go.jp (G.I.); 4 Graduate School of Frontier Sciences, The University of Tokyo, Chiba 277-8568, Japan; 5 Research and Services Division of Materials Data and Integrated System (MaDIS), National Institute for Materials Science (NIMS), Tsukuba 305-0044, Japan; 6 International Center for Young Scientists (ICYS), National Institute for Materials Science (NIMS), Tsukuba 305-0044, Ibaraki, Japan; MINAMI.Kosuke@nims.go.jp; 7 Center for Functional Sensor & Actuator (CFSN), Research Center for Functional Materials, National Institute for Materials Science (NIMS), Tsukuba 305-0044, Japan; NEMOTO.Takahiro@nims.go.jp; 8 Materials Science and Engineering, Graduate School of Pure and Applied Science, University of Tsukuba, Tsukuba 305-8571, Ibaraki, Japan

**Keywords:** Membrane-type Surface stress Sensor (MSS), artificial olfactory sensor, nanomechanical sensor, breath analysis, reproducibility, humidity

## Abstract

The endogenous volatile organic compounds (VOCs) in exhaled breath can be promising biomarkers for various diseases including cancers. An olfactory sensor has a possibility for extracting a specific feature from collective variations of the related VOCs with a certain health condition. For this approach, it is important to establish a feasible protocol for sampling exhaled breath in practical conditions to provide reproducible signal features. Here we report a robust protocol for the breath analysis, focusing on total expiratory breath measured by a Membrane-type Surface stress Sensor (MSS), which possesses practical characteristics for artificial olfactory systems. To assess its reproducibility, 83 exhaled breath samples were collected from one subject throughout more than a year. It has been confirmed that the reduction of humidity effects on the sensing signals either by controlling the humidity of purging room air or by normalizing the signal intensities leads to reasonable reproducibility verified by statistical analyses. We have also demonstrated the applicability of the protocol for detecting a target material by discriminating exhaled breaths collected from different subjects with pre- and post-alcohol ingestion on different occasions. This simple yet reproducible protocol based on the total expiratory breath measured by the MSS olfactory sensors will contribute to exploring the possibilities of clinical applications of breath diagnostics.

## 1. Introduction

Breath analysis has long been recognized as an ideal non-invasive diagnostic technique, which poses a potential for its future usage in disease detection and therapeutic monitoring [1]. Besides nitrogen, oxygen, carbon dioxide, and water vapor, human breath contains various kinds of volatile organic compounds (VOCs) [2] typically at the ppm to ppb range, while others may be even in ppt or lower concentration ranges [3]. Exhaled breath composition reflects the volatile compounds that are emitted from the membrane of the cells and/or from the surrounding microenvironment to the blood, although some volatile compounds originate in the airways, not being present in the blood [4]. Since some parts of VOCs in exhaled breath are produced endogenously through physiological or pathological processes, the composition of VOCs is believed to reflect a comprehensive metabolic state of the body. Indeed, several lines of evidence have revealed that certain types of VOCs could be related to various diseases [5]. It is thus rational to consider that patterns of VOCs in exhaled breath could be a predictive biomarker for disease detection. This notion has prompted many researchers to develop sensitive and reliable measurement techniques which are applicable to disease detection, especially early cancer detection [6]. Numerous studies have thus far reported that some exhaled VOCs are associated with cancer in terms of exhalation kinetics of VOCs [7], sensing response patterns of artificially intelligent nanoarrays [8], and diagnostic accuracy of breath tests [9], as well as specific correlations such as 15 VOCs for colorectal cancer [10], 3 VOCs for head and neck cancer [11], 12 VOCs for esophageal and gastric adenocarcinoma [12], 14 VOCs for breast cancer [13], and 22 VOCs for hepatocellular carcinoma [14]. However, clinical practice for measuring VOCs in exhaled breath has not been established yet. One of the major obstacles is that the majority of these approaches require a high level of expertise and expensive equipment such as gas chromatography-mass spectrometry (GC-MS) or various real-time analysis techniques including selected ion flow tube mass spectrometry and proton transfer reaction-mass spectrometry [15]. A simple and inexpensive artificial olfactory system, which detects multi-dimensional signals generated by multiple sensors array and analyses them by means of machine learning algorithm, has thus attracted much interest because of its applicability to on-site clinical applications as well as its ability to recognize the complicated patterns of VOCs as a whole.

A Membrane-type Surface stress Sensor (MSS) is a piezoresistive nanomechanical sensor which possesses potential as a sensing platform in an artificial olfactory system. The MSS consists of an adsorbate membrane suspended by four sensing beams, on which piezoresistors are embedded [16]. Response signals transduced by mechanical stress/strain, which is induced by sorption of gas molecules in the coating films coated on the membrane, exert more than 100-fold higher sensitivity in comparison with a standard piezoresistive cantilever-type sensor [17]. Recent studies have demonstrated the potential ability of MSS as an olfactory system in some different analysis, in settings such as quantification of alcohol contents from vapours of various types of liquids [18], quantification of a ternary mixture consisting of water, ethanol, and methanol [19], discrimination between spices and herbs [20] even with free-hand measurements [21], and detection of small amounts of VOCs contained in body odour [22]. To our interest, a pilot study analysing exhaled breath using MSS showed the possibility that patients with head and neck cancer can be discriminated from healthy volunteers [23] and patients before surgery from those after surgery as well as healthy control persons [24]. Although these previous studies have demonstrated promising sensitivity and specificity of MSS, it is known that the conditions of samples (e.g., temperature, humidity, and interfering gases) affect the signals measured by artificial olfactory systems based on chemical sensor arrays, including MSS. Thus, to assess the reproducibility and applicability of MSS olfactory sensors to the practical breath, a statistical evaluation of a large number of breath samples collected for a prolonged period is required.

In this study, we have conducted a statistical evaluation of breath samples collected throughout more than a year with controlled measurement conditions. For the breath sampling, we utilized so-called “total expiratory breath sampling” (also known as “mixed expiratory breath sampling”) [25], which is a simple method that can be practically implemented, thereby having a high potential to be standardized. In this method, the total exhaled breath is collected including dead-space air which is not involved in gaseous exchange in a lung. Although the other two major methods—“late expiratory breath sampling” and “end-tidal breath sampling”—can reduce the concentrations of exogenous gases by discarding the dead-space air, there is still no known optimal exclusion time or volume for breath analyses and diagnostics. In addition to the difficulties in controlling such conditions for each individual having distinct physiological properties, these methods may miss the airway gases that may contain an important breath marker, such as nitric oxide that has been adopted as the diagnosis and treatment of asthma [26]. Based on previous reports [4,25], the characteristics of these three methods are summarized in Table 1. The total expiratory breath contains all potential endogenous gases and there are a limited number of control parameters for the breath sampling, such as breath holding time, which can be rather easily controlled. Therefore, in this study, we focus on this simple approach and evaluate its reproducibility by analysing the sensing data collected throughout more than a year and the applicability to the detection of a testing material in exhaled breath using a same MSS standard measurement module. We have confirmed the high reproducibility of the present protocol achieved through the reduction of the major disturbances (i.e., interfering gases and humidity) in the total expiratory breath sampling by controlling measurement conditions and utilizing a simple data analysis method. As for the applicability of this breath measurement system, we demonstrated the detection of testing material, ethanol, in exhaled breath. Because of the simplicity and the possibility for standardization as well as the demonstrated reproducibility and applicability, the proposed artificial olfactory system based on the total expiratory breath measured by the MSS measurement module can be a promising candidate for future breath diagnostics.

## 2. Materials and Methods

### 2.1. Study Design

**Figure 1:**
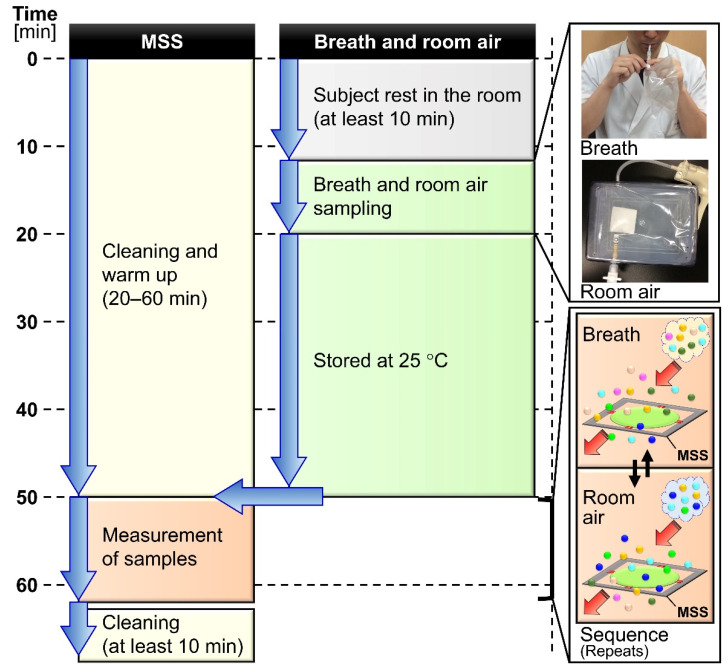
Flow chart of the exhaled breath assay with an outline of the protocol along the timeline. MSS stands for Membrane-type Surface stress Sensor.

For verifying the reproducibility of the proposed breath measurement system with various disturbances including the long-term drift of the sensing device and the seasonal variations of the ambient air, exhaled breath samples were repetitively collected in indoor environments from one male volunteer (age 34 to 36) on different days between July 2018 and December 2019 and all measured by the same MSS measurement module. For examining whether the MSS measurement module can detect a certain component in exhaled breath, ethanol was used as a testing material. Three male volunteers (age 34 to 42, mean 36.7) including one smoker, participated in the alcohol ingestion experiment between December 2018 and August 2019. Figure 1 shows the flow chart with the timeline in the experiments. This study was approved by the institutional review board of Ibaraki Prefectural Central Hospital, and informed consent was obtained from all individual participants.

### 2.2. MSS Receptor Layers and Measurement Module

**Figure 2:**
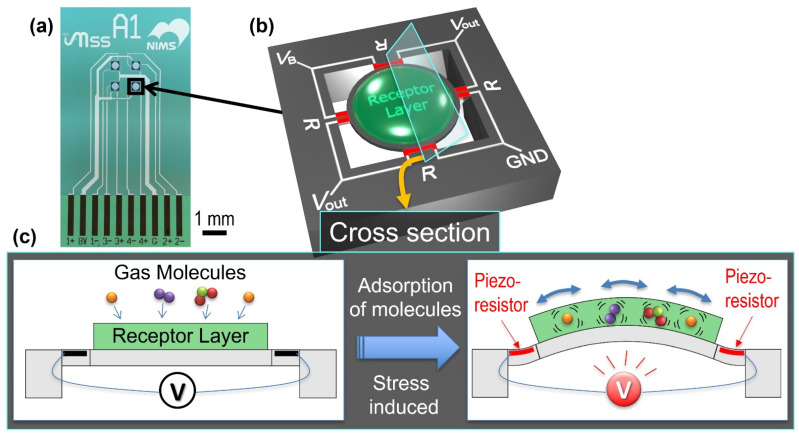
(**a**) Optical microscope image of an MSS chip. (**b**) Schematic illustration of an MSS chip with electrical connections. *R* indicates a piezoresistor, and *V*_B_, *V*_out_, and GND represent the connections to bridge voltage, output signal, and ground, respectively. (**c**) Working principle of MSS.

The MSS standard measurement module produced through the industry-academia-government framework called “MSS Alliance” and “MSS Forum” was used [27]. The working principle of MSS as well as its schematic illustration and an optical microscope image of an MSS chip are presented in Figure 2. The detailed fabrication process of an MSS chip is explained in previous reports [16,17]. The MSS chips used in the present study were purchased from NanoWorld AG. An inkjet spotter (LaboJet-500SP, MICROJET Corporation) equipped with a nozzle (IJHBS-300, MICROJET Corporation) was used to coat each receptor material. In this study, poly(4-methylstyrene), poly(2,6-diphenyl-1,4-phenylene oxide), and polymethyl methacrylate were used as receptor materials of Ch 1, 2, and 3, respectively. Each polymer was dissolved in *N,N*-dimethylformamide (DMF; 1 mg/mL), and the resulting solutions were deposited onto each channel of the MSS.

The MSS chips coated with the receptor layers were placed in a Teflon chamber, and the chamber was carefully sealed with O-rings. The chamber was connected to a gas flow system consisting of a switching valve connected with sampling and purging gas lines. The sample and purge gas flows were controlled by an aspiration pump with a flow rate adjusted to 30 mL/min. Data were measured at the bridge voltage of –1.0 V, and the relative resistance changes of piezoresistors were acquired at a sampling frequency of 100 Hz. Temperature and relative humidity (RH) of the sample and purge gases are monitored by a temperature/humidity sensor installed in the MSS measurement module. In the present study, the same module has been used for all the measurements without changing any components.

### 2.3. Sample Collection

Each exhaled breath sample was collected into a new 1000 mL polymer film sampling bag with a valve sleeve (Smart Bag PA; GL Sciences, Tokyo, Japan) using the following protocol. After 10 min of rest in the sampling room, the subject’s mouth was rinsed twice with 20 mL of normal saline. The subject inhaled and exhaled for 3 s each and repeated this cycle for three times (in total (3 + 3) × 3 = 18 [s] breathing) through the mouth, and inhaled for 2 s, and held the breath for 6 s, and then, slowly exhaled approximately 800 mL of the breath into the sampling bag through a polytetrafluoroethylene (PTFE) tube (50 mm length, 6 mm outer diameter and 3 mm inner diameter) (Flon Industry, Tokyo, Japan) via a connector (APU6; Nihon Pisco, Nagano, Japan). Along with breath sampling, room air in the breath sampling room was collected directly into another sampling bag by indirect negative pressure sampling to use as a purge gas to reduce the effects of the interfering gases. The obtained gas samples were stored in an incubator (SLC-25A; Mitsubishi Electric Engineering, Tokyo, Japan) at 25 °C. Each bag was disposed of after the measurement and was not reused.

### 2.4. Assay by the MSS Measurement Module

**Figure 3:**
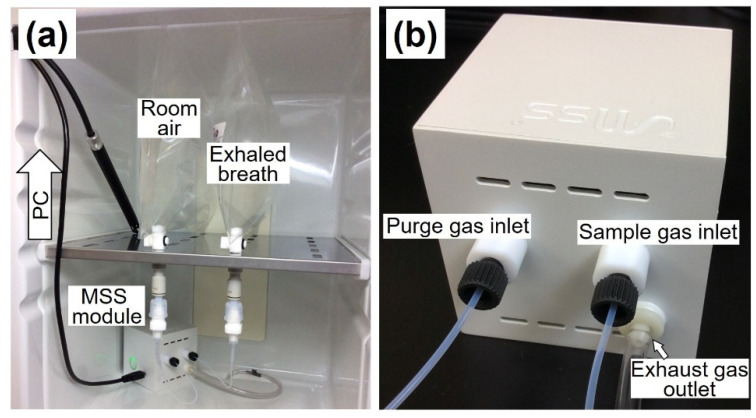
(**a**) Measurement setup for gas samples with the MSS measurement module. The module and gas samples were placed in an incubator set at 25 °C. (**b**) Inlet and outlet of gases on the module.

**Figure 4:**
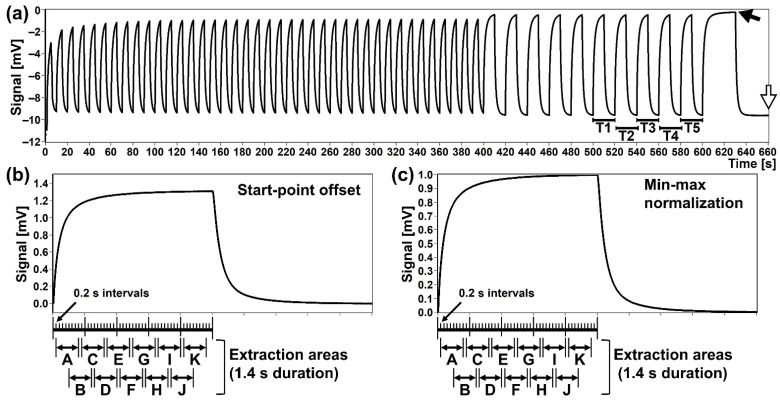
An example of an output waveform measured by the MSS measurement module. (**a**) A sequence of output waveforms. The last five of the 10 s cycles (denoted as T1–T5) were selected for the analysis. The black and white arrows indicate the measurement points of temperature and relative humidity (RH), in the sample and purge gases, respectively. (**b**,**c**) Examples of the output waveforms processed by the start-point offset method (**b**) and by the min-max normalization method (**c**). Data extraction areas were set as indicated by A–K.

All the measurements by the MSS measurement module were performed in an incubator kept at 25 °C. Immediately before the assay, the MSS measurement module was warmed up to 29–30 °C while flowing pure nitrogen gas (over 99.999% purity) through the sensors and gas flow lines. The sampling bags containing exhaled breath and the room air were stored at 25 °C for 30 min after the sampling. The sampling bags were connected to the MSS measurement module through the sample- and purge-injection lines, respectively (Figure 3a,b). To observe the dependence of the cycle time on the signals, the injection sequence was set with various cycle times as follows: 40 cycles of 5 s, 10 cycles of 10 s, and one cycle of 30 s exhaled breath sampling and room air purging (Figure 4a). Temperature and RH of the sample and purge gases were determined at the final cycle of the sequence using a temperature/humidity sensor in the MSS measurement module. For the exhaled breath samples in this study, the measured temperature and RH were almost constant within the ranges of 29.4–30.2 °C and 70.3–75.3%, respectively.

### 2.5. Verification of Reproducibility

Exhaled breath samples collected repetitively from one subject after at least 90 min fasting were analysed by the MSS measurement module. Headspace gas in an airtight vial containing sterile water was used as a reference, namely the humidified gas sample without interfering VOCs. In detail, nitrogen gas was bubbled into 10 mL of water through a PTFE tube (1/16-inch outer diameter, 1 mm inner diameter) which was connected to an aluminium bag (GL Sciences, Tokyo, Japan) pre-filled with nitrogen gas, and a headspace tube was directly connected to the sample injection line of the MSS measurement module. For the reference water headspace gas, it was confirmed that the measured temperature and RH in the MSS measurement module were almost constant within the ranges of 29.6–30.4 °C and 70.8–75.1%, respectively.

### 2.6. Detection of a Testing Material

After at least 90 min fasting, subjects took a dose of whiskey (40% *v*/*v*) equivalent to 0.5 g/kg body weight of ethanol in 30 min. Breath samples were collected at 0 and 60 min after the alcohol ingestion. During this experiment, subjects were allowed to take only water. Exhaled breath samples were analysed by both the MSS measurement module and the Kitagawa-type ethanol detector tube (104SB with AP-20 aspirating pump; Komyo Rikagaku Kogyo, Kanagawa, Japan), which detects 20–300 ppm of ethanol through the chemical reaction. This experiment was performed for three subjects and each subject participated on three different days.

### 2.7. Statistical Analysis

**Figure 5:**
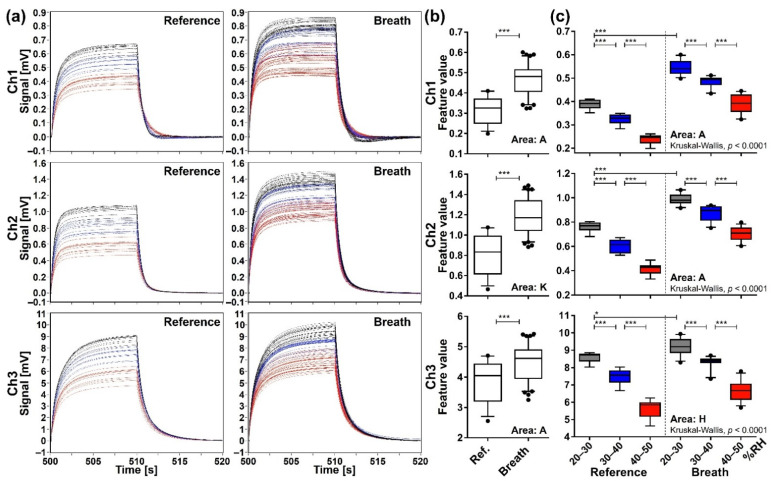
Sensing data processed by the start-point offset method. (**a**) The waveforms of reference (*n* = 33) and breath (*n* = 83) samples. (**b**) Comparison of feature values between reference and breath samples evaluated by the two-tailed Mann-Whitney U test (***, *p* < 0.0001). (**c**) Comparison of feature values among subgroups categorized by RH range of the purge gas: grey for 20–30% RH (reference *n* = 10, breath *n* = 24); blue for 30–40% RH (reference *n* = 12, breath *n* = 25); and red for 40–50% RH (reference *n* = 11, breath *n* = 34). Box plots represent the results of Kruskal-Wallis test and post-hoc Mann-Whitney U test with Bonferroni correction (*, *p* < 0.01; ***, *p* < 0.0001); horizontal lines, lower and upper edges of each box, and whiskers corresponds to medians, 25–75 percentile regions, and 5–95 percentile regions, respectively.

For analysing the responding signals measured by the MSS measurement module, the last five waveforms in the 10 s cycle area were used (Figure 4a). The waveforms were processed by both the start-point offset method and the min-max normalization method as shown in Figure 4b,c, respectively. In the start-point offset method, the signal value at the starting point of the sample gas measurement (e.g., at 500 s in Figure 5) is subtracted from the waveform data. The min-max normalization approach is processed to convert the response value of one waveform into a specific range. The signal data of a waveform were normalized by setting the minimum and maximum points to 0 and 1, respectively. The corrected signal data sets were then segmented into a predetermined size of the area with 50% overlap (Figure 4b,c, shown by A–K), as the sensors might respond with a different time constant to each odorous molecule. Data extraction areas of 1.4 s duration were set with 0.6 s overlap with the preceding extraction area for each of the waveforms. From each of the 1.4 s data extraction areas, 8 output signals with 0.2 s intervals were extracted, and the mean value of these 8 output signals was defined as the feature value of the extraction area. This analytical process was applied to each of the data extraction areas of the five waveforms which were generated by Ch 1–3 of the MSS.

To evaluate statistical significance between two sample groups, the Mann-Whitney U test was applied by using the EZR software version 1.41 (Saitama Medical Center, Jichi Medical University, Saitama, Japan). Two-tailed *p*-values of less than 0.01 were considered as statistically significant. The reproducibility of the assay was evaluated by the Bland-Altman analysis, which shows the bias (the mean difference between two feature values of measurements) and the 95% limits of agreement (bias ± 1.96 standard deviation (SD)). When zero in all extraction areas falls within the 30% confidence interval, namely 1 × SD of the bias, the assay was considered to be reproducible. This analysis is a graphical method, which is widely used to assess the reproducibility of continuous measures by plotting the difference scores of two measurements of the same variable.

The results were presented as a box plot or Bland-Altman plot using the GraphPad Prism version 6.03 software (GraphPad Software Inc., San Diego, CA, USA). In this report, we present the results obtained by analysing the T1 waveform, while we confirmed that the results were all reproduced in T2–T5 waveforms as well.

## 3. Results and Discussion

### 3.1. Overview of the Signals Measured by the Total Expiratory Breath Sampling Method with the MSS Measurement Module

Eighty-three exhaled samples and 33 reference water headspace gas samples were measured by the identical MSS measurement module. Figure 5a shows all the waveforms of exhaled breath and reference samples processed by the start-point offset method for each channel. It is found that the waveforms exhibit similar profiles specific to each sample/channel, whereas there are significant variations in the signal intensity. When these data are expressed by the feature values, the exhaled breath samples show significantly higher values compared to the reference (*p* < 0.0001) (Figure 5b), indicating that other components included in the breath samples in addition to water were detected. Although we showed only the extraction areas possessing the lowest *p*-value for each channel in Figure 5b, significant differences of feature values were observed exclusively in all extraction areas of Ch 1 and 2 and extraction areas A–F of Ch 3 (data not shown).

### 3.2. Effect of Humidity Variation in Room Air Used as a Purge Gas on the Sensing Signals

A sensing signal in a sample-purge injection cycle reflects the difference between the sample and purge gases, and thus the common gases included in both sample and purge gases have little effect on the sensing signal. Since most of exhaled breath is composed of the inhaled room air as well as a small portion of endogenous VOCs, it is effective to use room air as a purge gas to reduce the signals induced by uncertain interfering gases in room air, thereby enhancing the contributions of the endogenous VOCs to the sensing signal. Accordingly, as explained in the previous section, we used room air as a purge gas in the present study. In this case, however, it is difficult to reduce the contribution of humidity to the sensing signal because an exhaled breath is humidified when kept in a body (e.g., in an airway and a lung) at a certain range (in this assay, 71.2–75.3% for the measured 83 breath samples; cf. 70.8–75.1% for the measured 33 reference samples), while the humidity in room air can fluctuate in a much wider range (20.1–50.0% for the measured 83 and 33 samples).

To explicitly confirm the effect of humidity on the sensing signals, the reference and the exhaled breath samples were categorized into three subgroups according to the RH range in the purge gas, namely 20–30% (reference *n* = 10, breath *n* = 24), 30–40% (reference *n* = 12, breath *n* = 25), and 40–50% (reference *n* = 11, breath *n* = 34) subgroups. The overall difference among subgroups was evaluated by the Kruskal-Wallis test, which gave a *p*-value of less than 0.0001 for all extraction areas of each channel. Data of the extraction area with the lowest *p*-value are presented as representative data for each channel in Figure 5c. The Mann-Whitney U test with Bonferroni correction showed significant pairwise difference (*p* < 0.0001) among the RH subgroups if compared within the reference group or exhaled breath group. Significant differences were also observed between reference and exhaled breath samples if compared between the same RH subgroups (*p* < 0.0001 for Ch 1 and 2 and *p* < 0.01 for Ch 3). However, if compared irrespective of the RH subgroup (for example, comparison between 20–30% subgroup of reference in Ch 1 and 40–50% subgroup of breath), significant differences were not always observed between reference and breath samples. These results were consistent for all extraction areas of each channel (data not shown), indicating that the effect of RH in the purge gas cannot be ignored when interpreting the data processed by the start-point offset method.

It should be also noted here that the fluctuation of temperature is another major factor that possibly affects analytical results of exhaled breath, as reported previously [28]. However, the temperature of a sample is easier to control than the humidity by simply placing the sampling bags in a temperature-regulated incubator for a certain period (e.g., 30 min in the present study). In this study, the temperatures of the samples were well controlled within a rather small range (29.4–30.1 °C), which was confirmed not to induce a significant effect on the sensing signals.

### 3.3. Evaluation of Reproducibility through Humidity Correction and Signal Intensity Normalization

To evaluate how much reproducibility can be achieved by reducing the humidity effect, we utilized the following two approaches to reduce the effect of the humidity inconsistency between the sample and purge gases: (1) analysis of paired samples with close RH conditions and (2) normalization of signal intensity.

#### 3.3.1. Analysis of Paired Samples with Close RH Conditions

**Figure 6:**
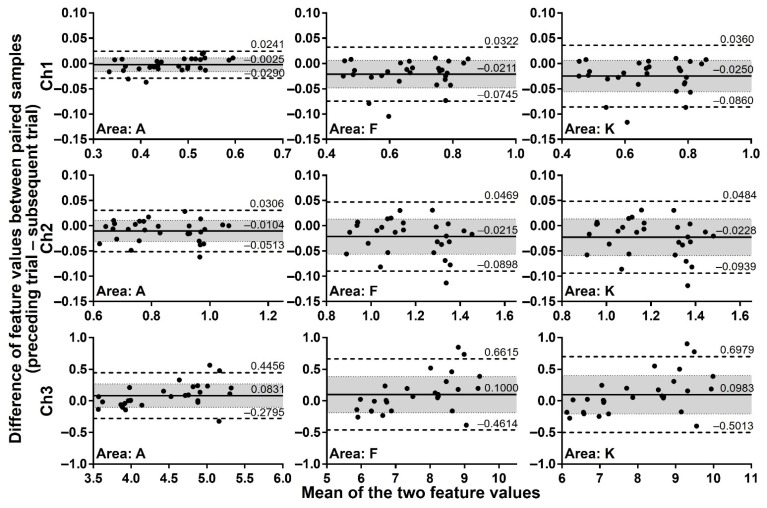
Difference of feature values between paired samples with the difference no more than 0.1% RH. The differences of feature values between 29 paired samples were evaluated by the Bland-Altman plots. Solid lines indicate the bias, and dashed lines correspond to 95% limits of agreement. Shaded areas represent 30% confidence intervals. The results of extraction areas A, F, and K are shown as representative plots.

To assess whether feature values of exhaled breath processed by the start-point offset method are reproducibly obtained if assayed under close RH conditions, paired samples collected under almost the same RH conditions (difference no more than 0.1% RH) were analysed by the Bland-Altman plots. Considering that breath samples, although collected from one same volunteer, are not identical because of biological variations of the body and effects of ingested foods, an allowable limit of the bias was set at 30% confidence intervals. Figure 6 depicts the Bland-Altman plots of all 29 paired samples for the extraction areas A, F, and K of Ch 1–3 as representative data. The analysis covering all extraction areas, namely areas A–K, showed that 89.7–93.1% of pairs for Ch 1 and 3 and 96.6% of pairs for Ch 2 are plotted within the 95% limits of agreement. The bias of the assay was from –0.0250 to –0.0025 for Ch 1, from –0.0228 to –0.0104 for Ch 2, and from 0.0831 to 0.1079 for Ch 3. Furthermore, the 30% confidence intervals always included the zero for all extraction areas of Ch 1–3, indicating that the measurements of the exhaled breath by the MSS measurement module exert high reproducibility when the humidity of room air used as a purge gas is well controlled at a certain value that is consistent in each assay.

#### 3.3.2. Normalization of Signal Intensity

It is usually challenging to control the humidity of room air in practical conditions. Thus, we also examined the possibility of reducing the humidity effect through the pre-treatment of the measured sensing data. For this purpose, we utilized the min-max normalization method. As this method compresses the waveform in between 0 and 1, the information on the signal intensity is mostly eliminated from the sensing data. Since the RH values of the purge gases (i.e., room air) correlate significantly with the signal intensity, as confirmed in Figure 5c, the min-max normalization method could reduce the humidity-related effect and relatively enhance the effects of remaining components, especially endogenous VOCs, included only in the breath samples.

**Figure 7:**
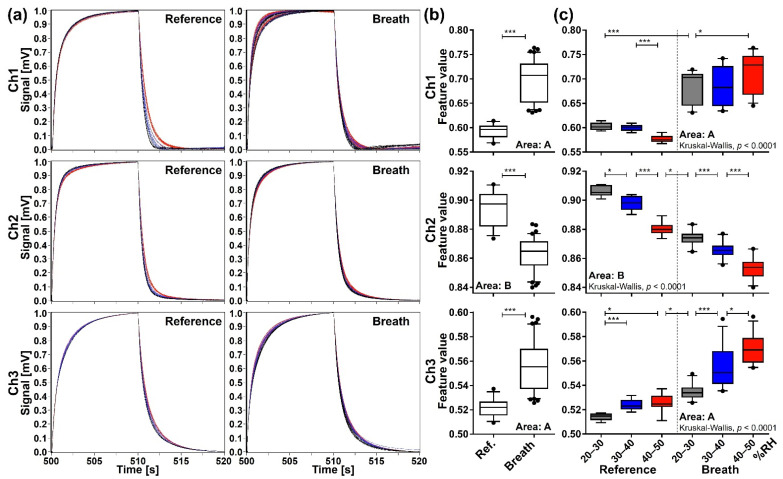
Sensing data processed by the min-max normalization method. (**a**) The waveforms of reference (*n* = 33) and breath (*n* = 83) samples. (**b**) Comparison of feature values between reference and breath samples evaluated by the two-tailed Mann-Whitney U test (***, *p* < 0.0001). (**c**) Comparison of feature values among subgroups categorized by RH range of the purge gas: grey for 20–30% RH (reference *n* = 10, breath *n* = 24); blue for 30–40% RH (reference *n* = 12, breath *n* = 25); and red for 40–50% RH (reference *n* = 11, breath *n* = 34). Box plots represent the results of Kruskal-Wallis test and post-hoc Mann-Whitney U test with Bonferroni correction (*, *p* < 0.01; ***, *p* < 0.0001).

The Kruskal-Wallis test showed an overall difference among each of the RH subgroups for all extraction areas of each channel (*p* < 0.0001). The data of the extraction area with the lowest *p*-value by the Kruskal-Wallis test are shown as representative data in Figure 7. Additional pairwise analysis by the Mann-Whitney U test with Bonferroni correction showed that feature values of exhaled breath samples are significantly different from those of reference samples (*p* < 0.01) irrespective of the RH subgroup in extraction area A–J of Ch 1, extraction area B, J, and K of Ch 2, and extraction area A, J, and K of Ch 3. These results suggest that the min-max normalization method can significantly reduce the effect of RH in the purge gas if appropriate extraction areas are selected, providing reproducible sensing data with certain information regarding the gaseous components included in breath samples.

### 3.4. Detection of a Testing Material in Exhaled Breath

**Figure 8:**
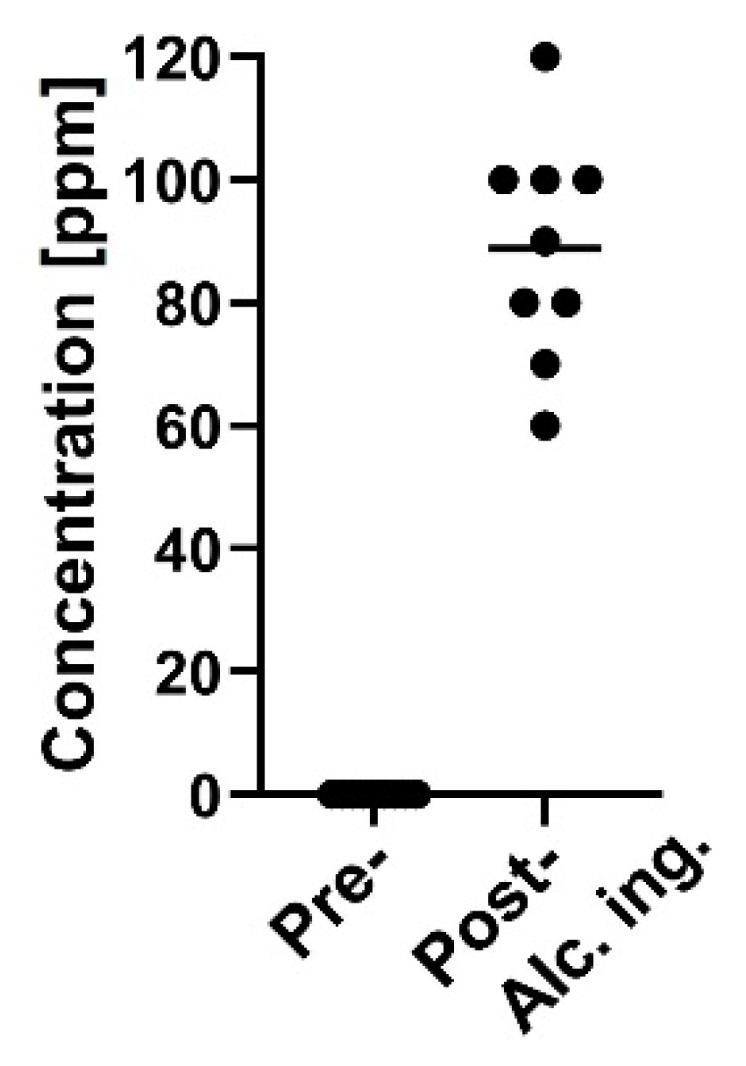
Breath ethanol concentration of pre- (*n* = 9) and post- (*n* = 9) alcohol ingestion. The mean value is represented as a horizontal line. Pre-alcohol ingestion samples exhibited undetectable levels (i.e., < 20 ppm).

**Figure 9:**
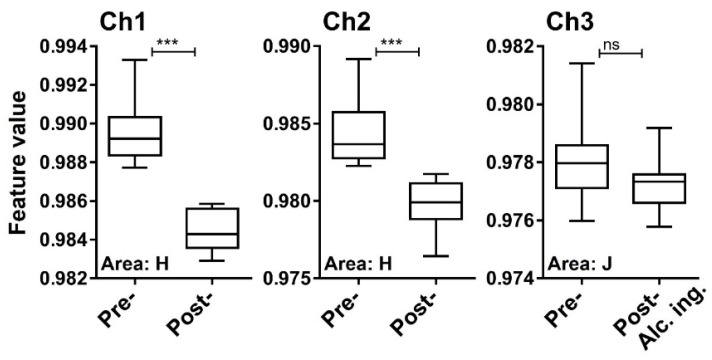
Feature values of pre- and post-alcohol ingestion breath samples processed by the min-max normalization method. Box plots represent the statistical differences evaluated by the two-tailed Mann-Whitney U test (***, *p* < 0.0001; ns, not significant).

We next examined whether a testing material, ethanol, in exhaled breath is detectable by the MSS measurement module. After one hour of alcohol ingestion, ethanol concentrations measured by the ethanol detector tube were in the range of 60–120 ppm (Figure 8). It was confirmed that in eighteen breath samples the measured temperature and RH in the MSS measurement module were constant within the ranges of 29.4–30.2 °C and 70.3–73.6%, respectively. For the room air, temperature and RH were observed within the ranges of 29.4–30.2 °C and 23.0–41.8%, respectively. As the humidity levels of room air used as purge gases were also not controlled in these measurements, significant differences in the feature values extracted from the sensing data processed by the start-point offset method were difficult to be observed between pre- and post-alcohol ingestion samples in all extraction areas of each channel. Thus, we applied the min-max normalization method to the obtained data and confirmed significant differences of feature values between pre- and post-alcohol ingestion samples in all extraction areas of Ch 1 and in extraction areas D–J of Ch 2. Contrary, a significant difference in the feature values was not observed between pre- and post-alcohol ingestion samples in all extraction areas of Ch 3. The extraction area with one of the lowest *p*-value for each channel is shown in Figure 9. These results are consistent with the previous observations that the receptor materials coated on Ch 1 and 2 are more effective in extracting the information on ethanol vapor because of their hydrophobic characteristics, in contrast to the receptor material on Ch 3 with the hydrophilic property [18]. These analyses suggest that the MSS measurement module with the appropriate data processing can discriminate testing material contained in exhaled breath, even if the breath samples were obtained from different examinees on different occasions with humidity variations.

## 4. Conclusions

In this study, we have established a simple yet robust protocol for breath analysis using the MSS olfactory sensor system. The reproducibility of the proposed breath measurement system based on the total expiratory breath sampling has been confirmed by the statistical evaluation of more than 80 exhaled breath samples collected from one volunteer throughout more than a year. It has been demonstrated that the key to achieving a reasonable reproducibility is to reduce the undesired effects, such as interfering exogenous gases and humidity, stemming from the differences between the sample and the purge gases. These two typical inconsistencies between sample and purge gases have been compensated by adopting the combination of the total expiratory breath sampling and the room air purge, and by suppressing the contributions of the humidity in a purge gas, respectively. Following the presented protocol, it has been confirmed that a testing material in exhaled breath is readily detectable. It should be noted that the main limitation of the present protocol for practical application is its rather long total assay time, which is about an hour as shown in Figure 1. Since the major parts of this assay include the warm-up operation of the measurement module and storage of breath sample, the development of an easy-to-use system with precise thermal control will significantly reduce the assay time as well as the total cost for each analysis.

While we have demonstrated the feasibility of assaying exhaled breath samples using the MSS olfactory sensor, this is a fundamental model experiment verifying the reproducibility and detecting a simple testing material in exhaled breath. Previous studies measuring exhaled breath of cancer patients suggest that diverse kinds of VOCs and their variations as a whole with up or down gradients of each component can be a predictive biomarker. Although the identification of a specific kind of a “marker molecule” will certainly be a breakthrough in breath diagnosis, the establishment of a “marker feature” in a pattern of multi-dimensional olfactory sensing signals will be another potential way to make a great leap towards practical implementation. In the latter approach, an easy and reproducible protocol with a feasible measurement device and controllable conditions is the essential requirement because such a “marker feature” must be reproduced by basically any operator irrespective of their expertise as well as the measurement conditions including the fluctuations of interfering gases and humidity. Although further investigations are required to assess the practical applicability and versatility of the presented assay, we believe that this simple protocol together with the MSS olfactory sensor technology can be a promising option. Moreover, this approach possesses plenty of room for further improvement in the performance through the optimization of receptor materials as well as the integration of advanced machine learning algorithms.

## Figures and Tables

**Table 1:** Characteristics of the three methods for collecting exhaled breath [4,25].

	Total Expiratory Breath (Mixed Expiratory Breath) *Applied in this Study*	Late Expiratory Breath	End-Tidal Breath (Alveolar Breath)
**Standardized procedure**	None	None	None
**Operation**	Simple	Relatively complicated	Relatively complicated
**Collecting exhaled breath phases**	All phases (Dead space + Transition + Alveolar)	Partial phases (Minimum dead space + Transition + Alveolar)	Partial phase (Alveolar)
**Discarding exhalation time**	None	First few seconds (Estimated dead space)	Initial portion (Monitoring CO_2_ level)
**Interfering exogenous volatile organic compounds**	Large	Moderate	Minimal

## Data Availability

The data presented in this study are available on request from the corresponding author. The data are not publicly available due to privacy restrictions.
